# PEX1^G843D^ remains functional in peroxisome biogenesis but is rapidly degraded by the proteasome

**DOI:** 10.1016/j.jbc.2025.108467

**Published:** 2025-03-28

**Authors:** Connor J. Sheedy, Soham P. Chowdhury, Bashir A. Ali, Julia Miyamoto, Eric Z. Pang, Julien Bacal, Katherine U. Tavasoli, Chris D. Richardson, Brooke M. Gardner

**Affiliations:** 1 Biomolecular Science and Engineering Program, University of California, Santa Barbara, Santa Barbara, California, USA; 2 Department of Molecular, Cellular, and Developmental Biology, University of California, Santa Barbara, Santa Barbara, California, USA; 3 Department of Ecology, Evolution, and Marine Biology, University of California, Santa Barbara, Santa Barbara, California, USA

**Keywords:** peroxisome, ATPases associated with diverse cellular activities (AAA), neurodegenerative disease, protein assembly, protein stability, protein degradation, proteasome, ubiquitin thioesterase (OTUB1)

## Abstract

The PEX1/PEX6 AAA-ATPase is required for the biogenesis and maintenance of peroxisomes. Mutations in *HsPEX1* and *HsPEX6* disrupt peroxisomal matrix protein import and are the leading cause of peroxisome biogenesis disorders. The most common disease-causing mutation in PEX1 is the *Hs*PEX1^G843D^ allele, which results in a reduction of peroxisomal protein import. Here, we demonstrate that the homologous yeast mutant, *Sc*Pex1^G700D^, reduces the stability of Pex1’s active D2 ATPase domain and impairs assembly with Pex6 *in vitro*, but can still form an active AAA-ATPase motor. *In vivo*, *Sc*Pex1^G700D^ exhibits only a slight defect in peroxisome import. We generated model human *Hs*PEX1^G843D^ cell lines and show that PEX1^G843D^ is rapidly degraded by the proteasome, but that induced overexpression of PEX1^G843D^ can restore peroxisome import. Additionally, we found that the G843D mutation reduces PEX1’s affinity for PEX6, and that impaired assembly is sufficient to induce degradation of PEX1^WT^. Lastly, we found that fusing a deubiquitinase to PEX1^G843D^ significantly hinders its degradation in mammalian cells. Altogether, our findings suggest a novel regulatory mechanism for PEX1/PEX6 hexamer assembly and highlight the potential of protein stabilization as a therapeutic strategy for peroxisome biogenesis disorders arising from the G843D mutation and other PEX1 hypomorphs.

Peroxisomes are membrane-bound organelles that are present in most eukaryotic cells (1). They harbor a dense matrix of enzymes to carry out unique metabolic reactions such as the beta-oxidation of very long chain fatty acids, the detoxification of reactive oxygen species , and the biosynthesis of plasmalogens contained in myelin (2, 3, 4, 5). Peroxisomes display a remarkable diversity of function: they participate in metabolic crosstalk with other organelles, innate immunity and antiviral signaling, and tissue-specific functions (6, 7, 8, 9). This adaptability suggests that cells can rapidly tune their peroxisome function according to environmental cues or pathogens. Owing to their roles in lipid metabolism and reactive oxygen species detoxification, peroxisomes are also essential for proper brain development and neurological function (10, 11).

The formation and maintenance of peroxisomes relies on ∼35 peroxins encoded by *PEX* genes (12). Mutations in at least 13 different *PEX* genes cause peroxisome biogenesis disorders (PBDs) that are characterized by widespread metabolic dysfunction (13). PBDs are divided into the broad group of Zellweger spectrum disorders (PBD-ZSDs) and the distinct rhizomelic chondrodysplasia punctata type 1 (14). Patients presenting with PBD-ZSD display a wide breadth of phenotype severity, ranging from developmental abnormalities and early infant mortality, to progressive sensorineural hearing loss and retinal degeneration (15). Due to the complex phenotypes presented by peroxisomal dysfunction, and the many genes associated with regulation of the organelle, a number of other disorders such as Usher syndrome are often misdiagnosed and later revealed to be PBDs through deep sequencing approaches (16). PBD-ZSD patients typically exhibit progressive deafness and retinopathy, but certain preventative measures such as diet restriction and avoidance of hepatotoxic substances have been shown to ameliorate symptoms. Therefore, strategies to improve peroxisomal protein targeting may be of broad benefit to PBD-ZSD patients, as well as the numerous other conditions in which peroxisome function is dysregulated.

The majority of PBD-ZSDs are caused by mutations in either *PEX1* or its binding partner *PEX6* (58.9% and 15.9%, respectively) (13). PEX1 and PEX6 assemble into a heterohexameric mechanoenzyme in the family of type-II AAA-ATPases, which contain two ATPase rings (17, 18). Proteins in this family use the energy of ATP hydrolysis to unfold substrates by processive threading or to disassemble protein complexes through large conformational changes, as exemplified by ClpB and human NSF, respectively (19, 20). The ATPase activity of PEX1/PEX6 is thought to drive the import of matrix proteins into the peroxisome by extracting the peroxisome targeting signal receptor PEX5 from the peroxisome membrane (21, 22, 23, 24). In the absence of PEX1/PEX6 function, PEX5 accumulates in the peroxisomal membrane (22), matrix protein import is impaired (25), and peroxisomes are targeted for peroxisome-specific autophagy (26, 27).

The most prevalent disease-causing mutation in PEX1, accounting for nearly 45% of observed cases, is a missense mutation converting glycine 843 to an aspartic acid (G843D) (28). Patients with the G843D mutation display milder phenotypes in the Zellweger spectrum and multiple lines of evidence suggest that PEX1^G843D^ is a hypomorphic allele (29). Patient fibroblasts homozygous for the G843D mutation exhibit a reduction of peroxisomal matrix protein import that can be partially restored by culturing cells at 30 °C (29, 30), by transfection with a plasmid expressing PEX1^G843D^ (31), and by overexpression of the PEX1 binding partner PEX6 (32). The G843D mutation reduces the level of PEX1 proteins in cells, a change that is not explained by a concomitant decrease in mRNA transcript levels (29, 33). Treatments that ameliorate the import defect in PEX1^G843D^ fibroblasts, such as growth at a reduced temperature of 30 °C, also increase PEX1 protein levels (29). Thus, the prevailing model in the field is that the G843D mutation causes a folding defect in PEX1 that destabilizes PEX1 and disrupts its interaction with PEX6 (29). However, it has not yet been shown how PEX1^G843D^ is degraded or how the G843D mutation impacts motor function. Mutations in PEX1 and PEX6 have been shown to induce peroxisome-specific autophagy (27), and there are conflicting reports regarding whether the inhibition of autophagy can improve peroxisome import in PEX1 hypomorphs (26, 34).

Recent advances in protein prediction and studies with the functionally homologous yeast Pex1/Pex6 complex suggest high structural conservation between experimentally determined yeast structures (35, 36) and AlphaFold-predicted human structures (37). These comparisons have allowed more accurate mapping of disease mutations and their proposed effects on PEX1/PEX6 function (38, 39). Similar to yeast Pex1/Pex6, human PEX1 and PEX6 each contain two N-terminal domains, N1 and N2, thought to be involved in substrate binding, and two ATPase domains, D1 and D2. The inactive D1 ATPase ring supports hexamerization, while the active D2 ATPase ring hydrolyzes ATP and drives substrate processing through conformational changes driven by ATP hydrolysis. The G843D mutation occurs at the transition between the D1-D2 linker and the beginning of the D2 ATPase domain. Given the location of the mutation and the well conserved nature of the glycine in this position, it is feasible that G843D alters D2 ATPase domain folding, ATPase activity, and/or assembly with PEX6. In this study, we set out to dissect how the G843D mutant impairs PEX1/PEX6 activity.

We utilize *in vitro* biochemistry techniques to demonstrate that the homologous G843D variant in yeast PEX1, *Sc*Pex1^G700D^, reduces the stability of the isolated D2 ATPase domain. We further find that the purified *Sc*Pex1^G700D^/*Sc*Pex6 hexamer displays residual ATPase and unfoldase activity, providing evidence for cooperative complex assembly in the presence of ATP. We then generate disease-model human cell lines and demonstrate that PEX1^G843D^ is rapidly degraded by the ubiquitin proteasome network, but otherwise capable of supporting peroxisome function *in vivo*. We additionally demonstrate that PEX1^G843D^ protein levels can be modestly stabilized by the knockdown of specific E3 ligases, and dramatically stabilized in mammalian cells when fused to a catalytically active deubiquitinase, OTUB1, suggesting that protein stabilization may be a promising therapeutic avenue. Finally, we find evidence that the stability of WT PEX1 is reduced in the absence of PEX6, indicating that orphan protein quality control mechanisms may fine tune the stoichiometry of the peroxisomal import machinery.

## Results

### Yeast Pex1^G700D^/Pex6 is a functional ATP unfoldase

**Figure 1:**
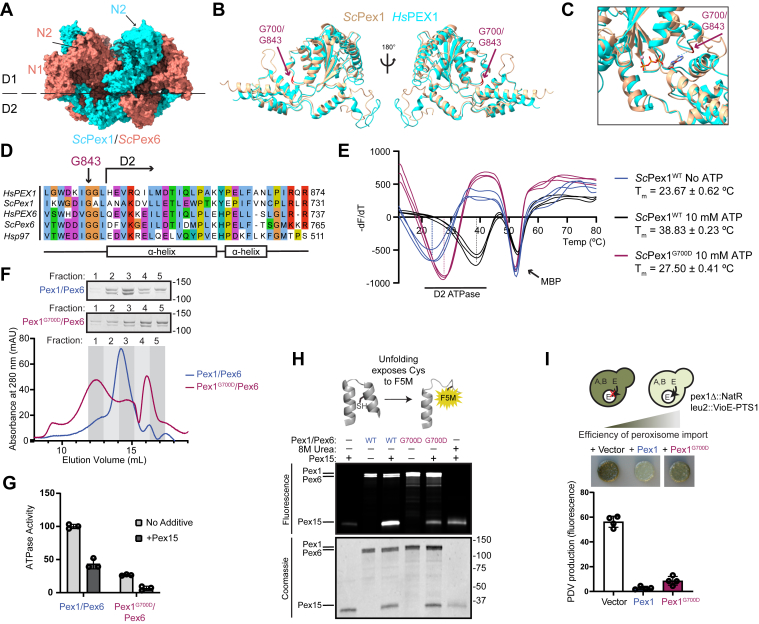
**Yeast Pex1^G700D^ is a partially active unfoldase *in vitro* but supports peroxisome import *in vivo***. *A*, structure and domain architecture of heterohexameric *Saccharomyces cerevisiae* Pex1/Pex6. PDB: 8U0V. *B*, structural alignment of AlphaFold2 predictions human (*cyan*) and yeast (*tan*) Pex1 D2 ATPase domains highlighting G843 and G700 residues (*red*) (37). *C*, as in B, focus on ATP binding pocket. ATP is modeled from AlphaFold2 structures based on alignment with human p97 (PDB: 7LN5) (74). *D*, sequence alignment of the G843 position in *Hs*PEX1 with *Sc*Pex1, *Hs*PEX6, *Sc*Pex6, and *Hs*p97. *E*, SYPRO Orange thermal melt assays of the MBP-D2 ATPase fusion. The plot shows the inverse derivative of raw RFU values for each replicate. Mean ± SD of N = 3 technical replicates. *F*, representative chromatograms of recombinantly purified *Sc*Pex1^G700D^/*Sc*Pex6 and *Sc*Pex1^WT^/*Sc*Pex6 on a Superose 6 Increase and Coomassie-stained SDS-PAGE of the fractions. *G*, ATPase assay using *S. cerevisiae* Pex1/Pex6 and Pex1^G700D^/Pex6 complexes and *Sc*Pex15 (1–309) recombinantly purified from *Escherichia coli*. Mean ± SD is shown from N = 3 technical replicates. *H*, fluorescein-5-maleimide (F5M) labeling experiments indicating *Sc*Pex1/*Sc*Pex6-mediated unfolding of *Sc*Pex15 (1–309). Structure (in *gray*) shows representative helices in *Sc*Pex15 (1–309) that contain buried cysteines that are labeled by F5M upon unfolding. Urea is a control for unfolding of *Sc*Pex15 without motor present. *I*, colorimetric assay for peroxisome import to measure complementation in a *Δpex1* deletion strain by *Sc*Pex1^G700D^ and *Sc*Pex1^WT^ with plate phenotype and quantification of prodeoxyviolacein pigment production. Mean ± SD is shown from N = 4 biological replicates. PDB, Protein Data Bank.

The pathogenic PEX1^G843D^ mutation replaces a glycine in the linker preceding the D2 ATPase domain with aspartic acid (Figs. 1, *A*–*C* and S1). Previous work has shown that recovery of peroxisome import in PEX1^G843D^ cells occurs at lower temperatures and upon addition of small molecule chaperones, both suggesting a defect in the folding of PEX1 caused by the G843D mutation (29, 30, 40, 41). As the glycine at this position is conserved from humans to yeast (Fig. 1*D*), and the predicted structure of this region of *Hs*PEX1 is highly similar to that of *Sc*Pex1 (Figs. 1, *B* and *C* and S1), we set out to assess how this mutation alters the folding of the yeast Pex1 D2 ATPase domain. We purified the isolated D2 ATPase domain as a maltose binding protein (MBP) fusion protein and measured domain stability using a SYPRO Orange thermal melt assay. We observed two unfolding events for the MBP-*Sc*Pex1^WT^ D2 ATPase fusion. One unfolding event observed at T_m_ = 52 °C was not sensitive to ATP and is presumably the MBP domain. Another unfolding event, presumably corresponding to the D2 ATPase domain was sensitive to the concentration of ATP: in the absence of ATP, unfolding was observed at T_m_ = 23.67 °C, while in the presence of 10 mM ATP, unfolding was observed at Tm = 38.8 °C (Fig. 1*E*). In comparison, we found that the G700D mutation reduced the melting temperature of the D2 ATPase domain to 27.5 °C from 38.8 °C for *Sc*Pex1^WT^ in the presence of 10 mM ATP (Fig. 1*E*). We were unable to observe temperature induced unfolding of *Sc*Pex1^G700D^ at lower concentrations of ATP. We therefore conclude that G700D destabilizes the isolated *Sc*Pex1 D2 ATPase domain, but that folding can be partially stabilized by nucleotide binding.

To test the functionality of the G700D mutant in the context of the heterohexameric Pex1/Pex6 complex, we purified recombinant *Sc*Pex1^G700D^/*Sc*Pex6 complexes. Using size-exclusion chromatography, we found that purified *Sc*Pex1^G700D^/*Sc*Pex6 reduced the proportion of Pex1/Pex6 in the hexameric fractions compared to the WT complex (Fig. 1*F*, fractions two and 3), and increased the proportion of slower migrating species, presumably Pex1 and Pex6 monomer or dimers (Fig. 1*F*, fractions 4 and 5, Fig. S2*A*). This shift indicates an increase in the rate of hexamer disassembly, or an assembly defect conferred by the G700D mutation. Despite this assembly defect, the *Sc*Pex1^G700D^/*Sc*Pex6 retained ∼30% ATPase activity compared to WT *Sc*Pex1/*Sc*Pex6 (Fig. 1*G*), and was also inhibited by the cytosolic domain of *Sc*Pex15 (Figs. 1*G* and S2*B*) (24). WT *Sc*Pex1/*Sc*Pex6 was previously shown to unfold truncated *Sc*Pex15 *in vitro* in a manner that depended on both active Pex1 and Pex6 D2 ATPase domains (24). Using a maleimide labeling assay for unfolding, in which we measure the labeling efficiency of buried cysteine residues as they become exposed, we found that the *Sc*Pex1^G700D^/*Sc*Pex6 complex unfolds truncated *Sc*Pex15, albeit with reduced efficiency compared to WT *Sc*Pex1/*Sc*Pex6 (Fig. 1*H*). These results suggest that although *Sc*Pex1^G700D^ assembly with *Sc*Pex6 is impaired, it retains the ability to form a functional AAA-ATPase that is capable of unfolding substrates.

To assess the *in vivo* effects of the mutation on Pex1/Pex6 function in *Saccharomyces cerevisiae*, we performed a complementation experiment in a *Δpex1* deletion strain harboring a colorimetric peroxisome import reporter. This strain is engineered to compartmentalize a key enzyme in the prodeoxyviolacein pathway to the peroxisome, such that when peroxisome import is impaired, the yeast produce a green pigment (Fig. 1*I*) (42). Reintroduction of *Sc*Pex1^G700D^ in a *Δpex1* deletion strain reduced green pigment formation nearly to the same levels as *Sc*Pex1^WT^, indicating that the *Sc*Pex1^G700D^ complements the *Δpex1* deletion with a mild defect (Fig. 1*I*). The observed import defect with the G700D mutation is therefore consistent with an import defect in patient fibroblasts expressing the G843D mutation (29). Taken together, we conclude that the G700D mutation partially compromises the folding of the Pex1 D2 ATPase domain and the assembly of hexamer with Pex6, but in the context of full-length Pex1, Pex6 binding partner, and high ATP concentrations, the *Sc*Pex1^G700D^ D2 ATPase domain can support peroxisome import function *in vivo*.

### The G843D mutation disrupts peroxisome import and decreases PEX1 protein levels in patient fibroblasts and in model cell lines

**Figure 2:**
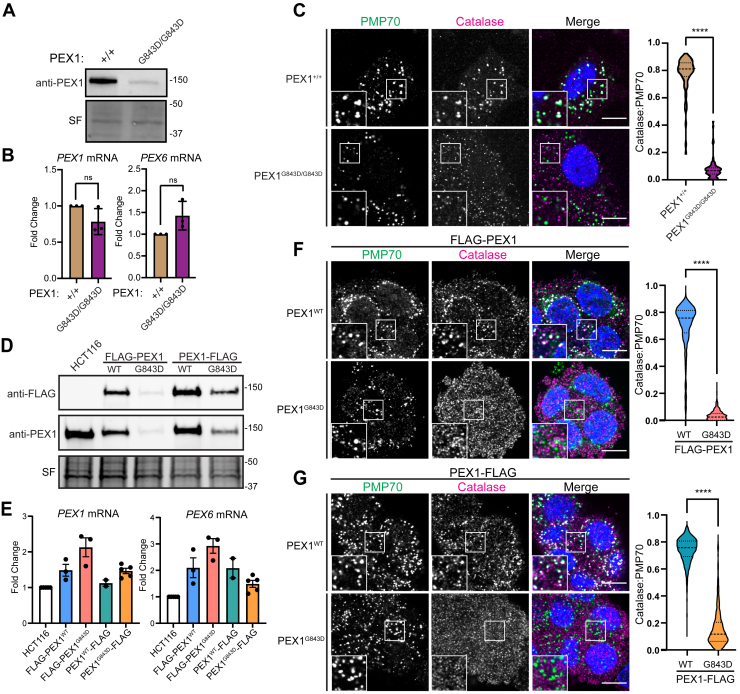
**Disease model HCT116 PEX1^G843D^ cell lines recapitulate PBD patient fibroblast phenotypes**. *A*, representative immunoblots of immortalized patient fibroblasts show decreased levels of PEX1^G843D^ (PEX1 = 142 kDa, SF = Stain-Free total protein). *B*, RT-qPCR in immortalized patient fibroblasts with genotypes of PEX1^+/+^ or PEX1^G843D/G843D^ indicate no significant change of *PEX1* and *PEX6* mRNAs. Mean ± SD is shown from N = 3 biological replicates, each done with N = 3 technical replicates. Unpaired *t* test with Welch's correction. *C*, representative images and quantification of PMP70/catalase colocalization from immunofluorescence microscopy of immortalized patient fibroblasts. Panels show PMP70 (*green*), catalase (*magenta*), and merged images with DAPI (*blue*) and insets show the colocalization of puncta calculated using Pearson’s colocalization coefficient on the pixel overlap of catalase and PMP70. The scale bar represents 10 μm. Unpaired *t* test with Welch's correction, *p*-value < 0.0001. *D*, representative immunoblots for anti-FLAG or anti-PEX1 in the HCT116 CRISPR-Cas9 edited clonal FLAG-PEX1/FLAG-PEX1, FLAG-PEX1^G843D^/FLAG-PEX1^G843D^, PEX1-FLAG/PEX1, and PEX1^G843D^-FLAG/PEX1^G843D^ cell lines. *E*, RT-qPCR in HCT116 FLAG-tagged PEX1 clonal cells indicate a slight increase in both *PEX1* and *PEX6* mRNA levels compared to unedited HCT116 cells. Mean ± SD is shown from N ≥ 2 biological replicates, each done with N = 3 technical replicates. *F*, as in (*C*) with HCT116 FLAG-PEX1/FLAG-PEX1 and FLAG-PEX1^G843D^/FLAG-PEX1^G843D^. The scale bar represents 10 μm. Unpaired *t* test with Welch's correction, *p*-value < 0.0001. *G*, as in (*C*) with HCT116 PEX1-FLAG/PEX1, and PEX1^G843D^-FLAG/PEX1^G843D^ cell lines. The scale bar represents 10 μm. Unpaired *t* test with Welch's correction, *p*-value < 0.0001. DAPI, 4′,6-diamidino-2-phenylindole; PBD, peroxisome biogenesis disorder; RT-qPCR, reverse transcription quantitative polymerase chain reaction.

Previous work in patient fibroblasts carrying the G843D mutation showed a strong reduction in the levels of PEX1 protein that was not explained by a concomitant reduction in mRNA levels. We confirmed markedly decreased PEX1 protein levels in immortalized and primary patient fibroblasts with the G843D mutation (Figs. 2*A* and S3*A*). The G843D fibroblasts had a modest, but statistically insignificant reduction in *PEX1* mRNA transcript levels and a slight increase in *PEX6* mRNA transcripts (Fig. 2*B*). As expected, PEX1^G843D/G843D^ cells presented a peroxisome import defect seen by significantly reduced colocalization of peroxisomal membrane protein PMP70 and peroxisomal matrix enzyme catalase (Fig. 2*C*). Due to the confounding effects of donor-to-donor variability in patient-derived cell lines, as well as the limitations of genetic tractability, we sought to create PEX1^G843D^ model cell lines to better isolate our study of the effects of PEX1^G843D^ (28, 43).

To create PEX1^G843D^ model cell lines, we first used Cas9 gene editing to create clonal homozygous N-terminal FLAG-PEX1 and heterozygous C-terminal PEX1-FLAG cell lines in human male colorectal carcinoma HCT116 cells. Into these two FLAG-tagged cell lines we used Cas9 gene editing to simultaneously introduce the G843D mutation and a silent mutation for a ClaI restriction enzyme cleavage site (Fig. S3*B*). We then screened clonal cells by ClaI digestion of PCR amplicons of the PEX1 gene from genomic DNA to identify clonal populations homozygous for PEX1^G843D^ (Fig. S3*C*).

In both FLAG-tagged cell lines, the introduction of the G843D allele significantly reduced PEX1 protein levels (Fig. 2*D*), recapitulating the reduced PEX1 abundance observed in patient fibroblasts. Analysis of *PEX1* and *PEX6* mRNA levels by reverse transcription quantitative polymerase chain reaction showed that introduction of the G843D mutation did not significantly alter *PEX1* or *PEX6* mRNA levels, similar to patient fibroblasts (Fig. 2*E*). Both FLAG-PEX1^G843D^ and PEX1^G843D^-FLAG cell lines exhibited reduced colocalization of peroxisomal membrane protein PMP70 and peroxisomal matrix enzyme catalase, recapitulating the import defect observed in patient fibroblasts (Fig. 2, *F* and *G*). Overall, the HCT116 cell lines with epitope tagged PEX1^G843D^ phenotypically resemble PEX1^G843D^ patient fibroblasts and provide a useful tool for interrogating the molecular effects of the G843D mutation.

### PEX1^G843D^ is rapidly degraded by the proteasome

**Figure 3:**
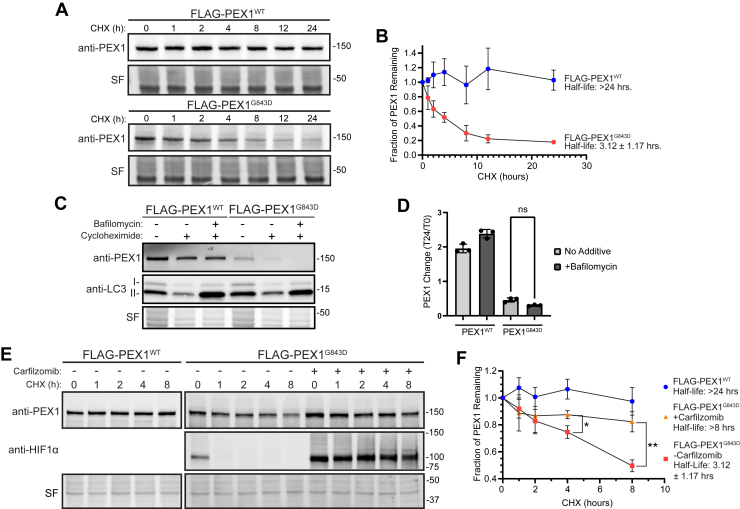
**PEX1^G843D^ is rapidly degraded by the proteasome and not autophagy**. *A*, representative immunoblots of cycloheximide (CHX) chase assay in FLAG-PEX1^WT^ and FLAG-PEX1^G843D^ clonal cell lines (PEX1 = 142 kDa, SF = Stain-Free total protein). *B*, quantification of CHX-chase assays shown in (*A*). Mean ± SD is shown from N = 4 biological replicates for each time point. *C*, representative immunoblots of PEX1 and LC3 in CHX-chase assay with the autophagy inhibitor Bafilomycin A1 (PEX1 = 142 kDa, LC3-I = 16–18 kDa, LC3-II = 14–16 kDa, SF = Stain-Free total protein). *D*, quantification of autophagy inhibition CHX-chase assays shown in (*C*). Mean ± SD is shown from N = 3 biological replicates for each time point. Unpaired *t* test with Welch’s correction. *E*, representative immunoblots of PEX1 and HIF1α in CHX-chase assay with proteasome inhibitor Carfilzomib (PEX1 = 142 kDa, HIF1α = ∼100 kDa, SF = Stain-Free total protein). *F*, quantification of proteasome inhibition CHX-chase assays shown in (*E*). Mean ± SD is shown from N = 3 biological replicates for each time point. Unpaired *t* test with Welch’s correction, *p*-values: ∗ = 0.02259, ∗∗ = 0.005724. CHX, cycloheximide.

The observed reduction of PEX1 protein without reduction of *PEX1* mRNA suggests that the G843D mutation either reduces PEX1 mRNA translation or PEX1 protein stability. To determine if the reduction of PEX1^G843D^ protein levels was due to increased degradation, we performed *in vivo* cycloheximide (CHX) chase experiments to compare the rate of degradation of FLAG-PEX1 and FLAG-PEX1^G843D^ homozygous HCT116 cell lines when uncoupled from the rate of translation. FLAG-PEX1 was stable over 24 h after translation was stalled with CHX, whereas FLAG-PEX1^G843D^ was rapidly degraded with a half-life of 3.12 ± 1.17 h (Fig. 3, *A* and *B*). Others have observed increased autophagic degradation of peroxisomes, or pexophagy, in patient cells with the G843D mutation, raising the question if pexophagy is responsible for PEX1^G843D^ degradation (26, 28, 29). We performed a similar experiment to test this hypothesis by culturing cells with either CHX, or CHX along with bafilomycin A1, an inhibitor of autophagosome and lysosome fusion, and therefore an inhibitor of autophagic degradation. The addition of autophagy inhibitor bafilomycin did not stabilize FLAG-PEX1^G843D^ in our CHX chase assay indicating that elevated autophagy activation was not the degradative mechanism for FLAG-PEX1^G843D^ (Fig. 3, *C* and *D*). We note that the autophagy inhibitor bafilomycin A1 resulted in an accumulation of LC3-II as expected, indicating that the treatment was effective at inhibiting autophagy. We then interrogated whether degradation was dependent on the proteasome. We found that the addition of the irreversible proteasome inhibitor carfilzomib greatly attenuated PEX1^G843D^ degradation with the half-life increasing from 3.12 ± 1.17 h to greater than the 8-h duration of the experiment (Fig. 3, *E* and *F*). Thus, the proteasome degrades PEX1 harboring the G843D mutation.

### PEX1^G843D^ can support peroxisome import function

**Figure 4:**
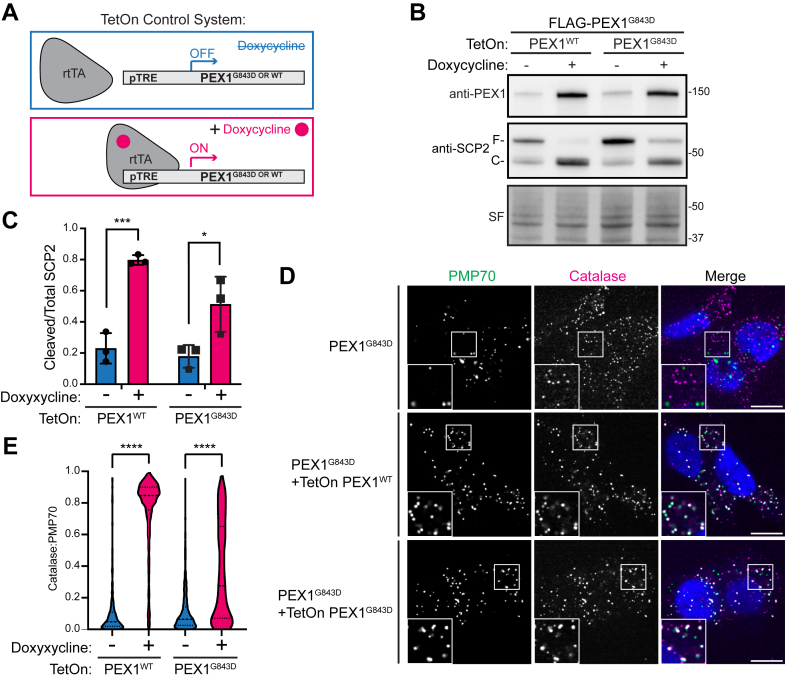
**Overexpression of PEX1^G843D^ partially rescues peroxisome function.***A*, schematic for the TetOn tunable gene expression constructs. Plasmid constructs contain a PEX1 variant expressed under Tet-responsive promoter (pTRE) and BFP-T2A-rtTA constitutively expressed under the hPGK promoter. Upon the addition of doxycycline, rtTA binds to pTRE to induce expression of PEX1 variants. *B*, representative immunoblots of PEX1 and SCP2 with doxycycline induced overexpression of both PEX1^WT^ and PEX1^G843D^ in FLAG-PEX1^G843D^ HCT116s (PEX1 = 142 kDa, SF = Stain-Free total protein). Full-length SCP2 (SCP2-F, 58 kDa) is cleaved upon import into the peroxisome (SCP2-C, 46 kDa). *C*, quantification of cleaved/total SCP2 upon induced expression of PEX1^WT^ or PEX1^G843D^ from replicates shown in (*B*). Mean ± SD is shown from N = 3 biological replicates. Unpaired *t* test, *p*-value summary: ∗∗∗ = 0.0007, ∗ = 0.0379. *D*, representative immunofluorescence microscopy images of catalase (*magenta*), PMP70 (*green*), and DAPI (*blue*) upon PEX1 overexpression. Insets show the colocalization of puncta. The scale bar represents 10 μm. *E*, quantification of catalase:PMP70 colocalization from experiments shown in (*D*), calculated using Pearson’s colocalization coefficient on the pixel overlap of catalase and PMP70. Unpaired *t* test with Welch's correction, all *p*-values < 0.0001. DAPI, 4′,6-diamidino-2-phenylindole

Our data in yeast showed that the homologous variant *Sc*Pex1^G700D^ could support peroxisome import *in vivo*, suggesting that PEX1^G843D^ may be functional if it was not targeted for degradation. To assess whether PEX1^G843D^ in human cells is similarly capable of supporting peroxisome import function, we used lentiviral transduction to stably introduce TetOn-PEX1^WT^ or TetOn-PEX1^G843D^ constructs into our FLAG-PEX1^G843D^ cell line to allow for tunable overexpression with doxycycline (Fig. 4*A*). The doxycycline-induced overexpression of PEX1^WT^ or PEX1^G843D^ was sufficient to restore PEX1 protein levels (Fig. 4*B*). We note that cells required higher concentrations of doxycycline to restore PEX1^G843D^ to WT levels. To assess whether peroxisome import function was restored by doxycycline induced overexpression, we used the proteolytic processing of SCP2 in the peroxisome as a readout. Full-length SCP2 (58 kDa) is cleaved to a smaller (46 kDa) active form upon peroxisomal import (44). Overexpression of PEX1^WT^ fully restored SCP2 processing whereas overexpression of PEX1^G843D^ strongly increased processing but was still partial compared to PEX1^WT^ (Fig. 4, *B* and *C*). Furthermore, when peroxisomal import was assessed by immunofluorescence microscopy, we found that PEX1^G843D^ overexpression strongly rescued peroxisomal import as seen by colocalization of a peroxisomal matrix protein catalase and a membrane protein PMP70. Interestingly, the overexpression of PEX1^G843D^ produced a mosaic of rescue phenotypes compared to the full rescue with PEX1^WT^ overexpression (Fig. 4, *D* and *E*). This observation highlights the strong propensity for PEX1^G843D^ to be degraded by the proteasome and suggests the existence of a “critical threshold” of PEX1^G843D^ that can fully rescue the import defect. Taken together, these data suggest that PBDs arising from the G843D mutation can be ameliorated by stabilizing PEX1^G843D^ protein levels.

### Assembly defect of the PEX1/PEX6 complex promotes degradation of PEX1

**Figure 5:**
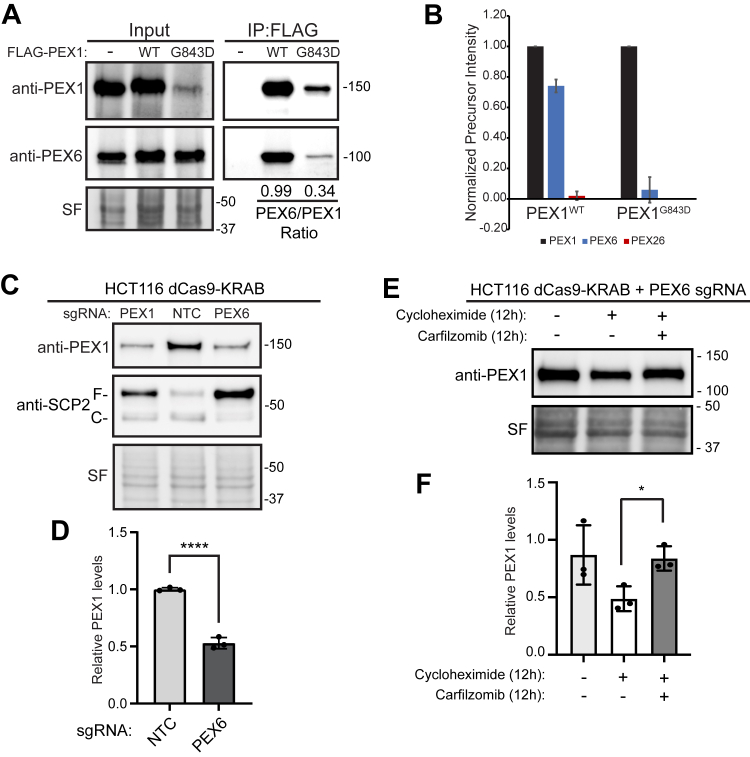
**An assembly defect is sufficient to cause degradation of PEX1^WT^ and PEX1^G843D^**. *A*, immunoprecipitation of FLAG-PEX1 and FLAG-PEX1^G843D^ from clonal HCT116s show reduced coimmunoprecipitation of PEX6 in FLAG-PEX1^G843D^ cell lines (PEX1 = 142 kDa, PEX6 = 106 kDa, SF = Stain-Free total protein). Quantification of the PEX6/PEX1 ratio from the immunoblots is shown below each respective lane in the IP:FLAG elutions for PEX1^WT^ and PEX1^G843D^. *B*, mass spectrometry of eluates from FLAG immunoprecipitations in FLAG-PEX1^WT^ and FLAG-PEX1^G843D^ cell lines shows a significant decrease in abundance of PEX1 binding partners PEX6 and PEX26 with the G843D mutation. Total precursor intensity was normalized to PEX1 total precursor intensity levels. Mean ± SD of two biological replicates (Table S1). *C*, knockdown of PEX6 with sgRNA in dCas9-KRAB expressing HCT116 cells causes degradation of PEX1^WT^ and phenocopies a PEX1 knockdown (PEX1 = 142 kDa, SF = Stain-Free total protein). Full-length SCP2 (SCP2-F, 58 kDa) is cleaved upon import into the peroxisome (SCP2-C, 46 kDa). *D*, quantification of PEX1 levels in PEX6 knockdown and NTC cells from (*C*) show a significant decrease in PEX1^WT^ levels. Mean ± SD is shown for N = 4 biological replicates. Unpaired *t* test, *p*-value <0.0001. *E*, representative immunoblots of PEX6 knockdown in HCT116-dCas9-KRAB cells treated with either cycloheximide alone or cycloheximide and carfilzomib for 12 h. *F*, quantification of PEX1 levels from (*E*) show a significant stabilization in PEX1^WT^ levels in the presence of carfilzomib. Data are normalized to PEX1 levels in untreated NTC cells. Mean ± SD is shown from N = 3 biological replicates. Unpaired *t* test with Welch's correction, *p*-value ∗ = 0.016. NTC, nontargeting control

It has previously been shown that the G843D mutation reduces PEX1 binding to PEX6 (32, 45). To test if the *Hs*PEX1^G843D^ mutation impairs assembly with *Hs*PEX6 in the HCT116 cell lines, we immunoprecipitated PEX1^G843D^ from both the N and C terminally FLAG-tagged cell lines and identified coimmunoprecipitating proteins by immunoblotting and mass spectrometry (MS). Immunoblotting of the FLAG-PEX1 coprecipitants showed significantly less enrichment of PEX6 copurified with PEX1^G843D^ compared to PEX1^WT^ (Figs. 5*A* and S4*A*). MS of both FLAG-tagged PEX1 cell lines showed significantly reduced abundance of PEX1 binding partners PEX6 and PEX26 when G843D was present, confirming that PEX1^G843D^ impairs assembly with PEX6 (Figs. 5*B* and S4*B*).

Recent studies have demonstrated that cells degrade unpaired, or “orphan”, subunits of multiprotein assemblies in an attempt to regulate protein complex stoichiometry (46). To determine whether the assembly of the PEX1/PEX6 complex is also subject to orphan protein quality control mechanisms, we tested if depletion of PEX1’s binding partner PEX6 is sufficient to induce the degradation of PEX1^WT^. We found that knockdown of PEX6 in HCT116 CRISPRi (dCas9-KRAB) (47, 48, 49) cells significantly reduced WT PEX1 protein levels (Figs. 5, *C* and *D* and S4, *C* and *D*). To confirm whether the loss of PEX1^WT^ in PEX6 depleted cells was due to proteasomal degradation of PEX1, we performed a CHX chase experiment to assess the stability of PEX1^WT^ in PEX6 depleted conditions. We observed that PEX1 levels were reduced by 12 h in CHX treatment alone and stabilized when cells were cotreated with CHX and carfilzomib (Fig. 5, *E* and *F*). Consistent with PEX1 degradation by the proteasome, large-scale proteomic studies identified ubiquitination modifications at over 30 different lysine residues in PEX1 (50, 51, 52, 53, 54). These data suggest that the PEX1/PEX6 complex is subject to a previously uncharacterized orphan protein quality control mechanism, and that WT PEX1 is degraded by the proteasome in the absence of its PEX6 binding partner. Therefore, we conclude that PEX1^G843D^ is defective in its ability to bind PEX6, which exacerbates PEX1 orphan protein quality control, likely in addition to other redundant misfolded protein quality control pathways that target PEX1^G843D^ for degradation.

### PEX1^G843D^ is stabilized by inhibiting ubiquitination

**Figure 6:**
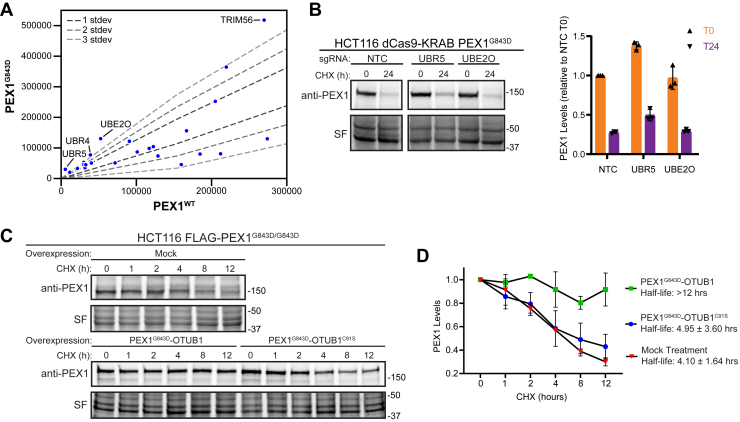
**PEX1^G843D^ degradation is hindered by fusion to a deubiquitinase OTUB1**. *A*, ubiquitin ligase related proteins identified by anti-FLAG copurification MS/MS in FLAG-PEX1^WT^, FLAG-PEX1^G843D^, PEX1^WT^-FLAG, and PEX1^G843D^-FLAG cell lines. The plot shows the comparison between WT and G843D samples on a subset of total identified proteins related to ubiquitin interacting proteins. Comparisons are the mean of total precursor intensity from two WT and two G843D samples (Table S2). *B*, representative immunoblots and quantification of PEX1 levels of PEX1^G843D/G843D^ HCT116 CRISPRi (dCas9-KRAB) cell lines with knockdown of UBR5 and UBE2O candidates identified from the IP-MS experiment (PEX1 = 142 kDa, SF = Stain-Free total protein). Knockdown of mRNA transcripts verified in Fig. S5. Cells were treated with cycloheximide at T = 0 and samples collected after 24 h. Mean ± SD is shown from N = 3 biological replicates for each timepoint. *C*, representative immunoblots of PEX1-OTUB1 fusions in a CHX-chase assay. OTUB1^C91S^ is catalytically inactive. *D*, quantification of replicate experiments similar to (*C*). Mean ± SD is shown from N = 3 biological replicates for each time point. CHX, cycloheximide; IP-MS, immunoprecipitation mass-spectrometry.

Several E3 ligases have been recently identified to monitor the formation of protein complexes and to ubiquitinate orphan proteins that have not properly assembled, such as UBE2O, HUWE1, UBR5, and SCF^Das1^ (55, 56, 57, 58). In our coimmunoprecipitation MS, we identified multiple E3 ligases enriched in binding to PEX1^G843D^ compared to PEX1^WT^ (Fig. 6*A*). We generated HCT116 CRISPRi (dCas9-KRAB) (47, 48, 49) cells homozygous for the G843D mutation using the same strategy as for the FLAG-PEX1 cell lines (Fig. S5, *A* and *B*). We then knocked down two E3 ligases that were enriched in FLAG-PEX1^G843D^ eluates (Fig. 6*A*) and have been implicated in orphan protein quality control, UBR5 and UBE2O (56, 58). We observed a modest increase in the steady state levels of PEX1 in UBR5 knockdowns, as well as a modest increase in the levels of PEX1 protein remaining 24 h after CHX treatment (Fig. 6*B*). UBE2O knockdown appeared to have no effect on PEX1 protein levels (Fig. 6*B*), although the knockdown of *UBE2O* mRNA was effective (Fig. S5*C*). These experiments suggest that UBR5 may be involved in ubiquitination of PEX1^G843D^, and that knockdown of this ligase can modestly stabilize PEX1^G843D^. We expect PEX1^G843D^ to be targeted by several E3 ligases since it has both a folding and assembly defect, so it is unsurprising that PEX1^G843D^ levels do not recover to WT levels upon knockdown of a single E3 ligase.

To further establish that PEX1^G843D^ is targeted to the proteasome by ubiquitination and to rule out the possibility that PEX1^G843D^ is degraded by a proteasome-dependent, yet ubiquitination-independent mechanism (59), we tested if direct fusion of PEX1^G843D^ to a catalytically active deubiquitinase could shield PEX1 from degradation. This OTUB1-deubiquitinase fusion is similar to the deubiquitinase-targeted chimera (DUBTAC) strategy that has been shown to effectively protect proteins from overzealous protein degradation (60). We generated constructs to express a chimera of PEX1^G843D^ with the deubiquitinase OTUB1 fused to the C terminus of PEX1^G843D^. We transfected these constructs into the FLAG-PEX1^G843D^ cells and performed a CHX chase assay. We found that PEX1^G843D^-OTUB1 was stable over the 12 h course of the assay (Fig. 6C). Critically, an identical chimeric construct of PEX1^G843D^ with catalytically inactive OTUB1, OTUB1^C91S^, was not stabilized in this assay (Fig. 6*C*). Densitometry quantification of replicate immunoblot experiments show that PEX1^G843D^-OTUB1 was significantly stabilized while PEX1^G843D^-OTUB1^C91S^ was degraded at a rate similar to endogenous PEX1^G843D^ (Fig. 6*D*). Altogether, our results suggest that degradation of PEX1^G843D^ is ubiquitin-dependent, and that targeted deubiquitination is sufficient to protect PEX1^G843D^ from proteasomal degradation.

## Discussion

In this study, we leveraged the tractability of the yeast Pex1/Pex6 proteins and Cas9 gene editing in human cell lines to explore the consequences of the G843D mutation on PEX1. Our *in vitro* biochemical analysis of the homologous yeast variant, *Sc*Pex1^G700D^, showed that this mutation directly destabilizes the D2 ATPase domain and reduces assembly with Pex6. Despite these defects, *Sc*Pex1^G700D^/Pex6 retains ATPase and unfoldase activity *in vitro*. Additionally, expression of Pex1^G700D^ mostly restored peroxisome import in the Δ*pex1* strain, suggesting that Pex1^G700D^ is functional when overexpressed. In human cells, we found that PEX1^G843D^ was subject to rapid degradation by the ubiquitin proteasome system, but when overexpressed, PEX1^G843D^ could support peroxisome function. Thus, our data show that PEX1^G843D^ retains function, but is degraded by overzealous protein quality control machinery. This work suggests that the widely varying clinical phenotypes and disease severity of patients harboring PEX1^G843D^ depends on the residual levels of PEX1 protein and is sensitive to variations in the ubiquitin-proteasome system. Future work could explore the synergistic effects of treatments that stabilize PEX1^G843D^ alongside small molecules that have been shown to improve peroxisome import in PEX1^G843D^ cell lines (40, 41, 61).

Protein quality control by ubiquitin-mediated degradation is a well-established posttranslational regulatory mechanism. Recent studies have shown that cells not only monitor protein folding, but also the correct assembly of large protein complexes. Indeed, for some AAA-ATPases, such as the proteasome, assembly is a strictly regulated process with co-chaperones and assembly factors that facilitate proper pairing or degradation of each AAA-ATPase domain (62, 63). From our experiments, we surmise that PEX1^G843D^ degradation occurs in a complex manner with recognition by multiple components of the ubiquitin-proteasome system due to both intrinsic folding instability and poor complex formation with PEX6. Thus, the functional redundancy between E3 ligases precludes a strong phenotype from single gene knockdowns, but forced proximity of a catalytically active deubiquitinase clearly protects the protein from ubiquitin-proteasome dependent degradation. Interestingly, we found that even WT PEX1 was subject to proteasomal degradation in the absence of its binding partner PEX6. This observation suggests that assembly of PEX1 and PEX6 masks a previously unknown “degron” in PEX1, and thus there may be other factors that specifically monitor PEX1/PEX6 assembly that are yet to be identified. Therefore, the degradation of orphaned PEX1 appears to be important for cellular function. Since PEX6 binds the peroxisomal anchoring protein, PEX26, orphaned PEX1 lacks the information for localization to the peroxisome and may mislocalize and interact with other Type 2 AAA-ATPases such as p97, SPATA5, and ATAD2 which were also identified as coimmunoprecipitants in our PEX1 MS experiments. AAA-ATPases may be prone to mismatch since the conservation of the nucleotide binding sites at the interfaces of subunits likely restricts variation and specialization in these interfaces.

Understanding the proteasome-dependent regulation of the PEX1/PEX6 complex could unveil new strategies for improving clinical outcomes in patients with PEX1^G843D^ or other mutations in PEX1 that result in protein level changes and milder phenotypes. While an ideal treatment would be the systemic reintroduction of PEX1^WT^ as a gene therapy, such an approach remains impractical at postembryonic stages. Instead, strategies to stabilize the residual protein levels of PEX1 hypomorphs could be modeled on promising approaches to treating other diseases exacerbated by overzealous protein quality control such as cystic fibrosis. The key steps in the development of these therapeutic strategies thus include the identification of small molecules that stabilize PEX1 protein folding or PEX1/PEX6 assembly, as well as discovery of small molecule ligands that bind PEX1, which can then be conjugated to small molecules that recruit endogenous deubiquitinases to the target (60). While this study used the common disease-causing allele PEX1^G843D^, many of the rare alleles in PEX1 and PEX6 also known to cause disease are associated with reduced assembly of PEX1/PEX6 and reduced levels of PEX1 or PEX6 proteins (32). Additionally, studies suggest that import into peroxisomes declines with age and that peroxisome biogenesis is modulated by viruses (64, 65, 66). Thus, strategies that improve PEX1/PEX6 function and peroxisome import may improve human health beyond PEX1^G843D^ -associated PBDs.

## Experimental procedures

### Purification of recombinant proteins

Pex1-FLAG and His-Pex6 WT and G700D complexes were coexpressed in BL21 *Escherichia coli* from the pETDuet and pCOLADuet vectors, respectively. His-MBP-PSP-Pex1 WT and G700D D2 domains were expressed in BL21 *E. coli* from the pCOLA vector. The expression strains were grown in DYT (16 g tryptone, 10 g yeast extract, and 5 g NaCl) or DYT with 0.2% dextrose (for His-MBP-PSP-Pex1 D2 domain constructs) with appropriate antibiotics at 30 °C. Cultures were induced at *A*_600_ = 0.6 to 0.8 with 0.3 mM IPTG before overnight incubation at 18 °C or 3-h incubation at 30 °C for Pex1 D2 domain constructs. The *E. coli* were harvested at 4000*g* 20 min at 4 °C, and the pellet was resuspended in NiA buffer (30–50 mM Hepes pH 7.6, 100 mM NaCl, 100 mM KCl, 10% glycerol, 10 mM MgCl_2_, 20 mM imidazole, and 1 mM ATP (VWR, #97061–226)) with lysozyme (0.2 mg/ml, MP Bio, #02100831-CF) and protease inhibitors: leupeptin, pepstatin, aprotinin, and PMSF. Cells were lysed by sonication at 60% amplitude with 15 s pulses on 90 s off for a total of 180 s on. The total lysate was spun at 30,000*g* 30 min at 4 °C to remove insoluble cell debris, and the supernatant was transferred to conical tubes with 5 ml of preequilibrated Ni-NTA agarose (Thermo Fisher Scientific, #88223). The cell lysate and Ni-NTA agarose were incubated with inversion at 4 °C for 1 h before the Ni-NTA agarose was batch-washed twice with 50 ml NiA buffer. The Ni-NTA agarose was resuspended in NiA and poured into gravity flow columns and washed until the flowthrough contained no protein, as judged by Bradford assay. The bound protein was then eluted with NiA containing 500 mM imidazole. For the Pex1-FLAG/His-Pex6 complexes, the Ni-NTA eluate was then added to preequilibrated anti-FLAG M2 agarose affinity resin (Sigma-Aldrich, #A2220). The anti-FLAG resin was equilibrated by first washing with two sequential column volumes of GF buffer (50–60 mM Hepes pH 7.6, 50 mM NaCl, 50 mM KCl, 5% glycerol, 10 mM MgCl_2_, and 1 mM ATP), then with three sequential column volumes of 100 mM glycine HCL pH 3.5, followed by equilibration with five sequential column volumes of GF. The Ni-NTA eluate was passed over the FLAG-resin twice to bind, and then was washed with GF buffer until flowthrough contained no protein, as judged by Bradford assay. The bound protein was eluted with GF and 300 μg/ml FLAG peptide (Sigma-Aldrich, #F3290) and subsequently concentrated on a spin concentrator (30 or 100 MWCO, Millipore Sigma, #UFC910008) before snap-freezing in liquid nitrogen and storing at −80 °C. To separate Pex1-FLAG/His-Pex6 hexamer from other oligomers and monomers by FPLC, the concentrated FLAG eluates were loaded onto a Superose-6 size-exclusion column (Cytiva, #29091596) equilibrated in GF buffer. For the complex assembly analysis of WT and G700D complexes, equal concentrations of concentrated FLAG-elution were loaded on the FPLC and fractions of different peaks were analyzed by SDS-PAGE and Coomassie-blue staining. The concentration of protein was determined with a Bradford assay.

For His-MBP-PSP-Pex1 D2 domain constructs, the same protocol was followed through the Ni-NTA elution, where the resulting eluate was batch bound to amylose agarose resin rather than FLAG affinity resin and eluted with NiA buffer and 10 mM maltose. Eluted protein was concentrated on a spin concentrator (30 MWCO, Millipore Sigma, #UFC903008). To further purify His-MBP-PSP-Pex1 WT and G700D D2 domains, the concentrated amylose eluates were loaded onto a Superdex 200 (Cytiva #28990944) size-exclusion column in NiA buffer. Fractions of different peaks were analyzed by SDS-PAGE and Coomassie-blue staining to identify isolated His-PSP-MBP-Pex1 D2 domains. Identified fractions were subsequently concentrated on a spin concentrator (30 MWCO), snap-frozen in liquid nitrogen, and stored at −80 °C. Protein concentration was determined *via* Bradford assay.

To purify the cytosolic domain of *Sc*Pex15 (amino acids 1–309), we replaced the transmembrane domain and cytosolic amphipathic helix with a FLAG-6xHis and an HRV-3C PreScission protease (PSP) site between Pex15 and the affinity tags. This construct was purified with the same protocol as for Pex1/Pex6 up to the Ni-NTA elution, except without ATP in the buffers. The Ni-NTA eluate was then concentrated with spin concentrators, and HRV-3C PreScission protease (GenScript #Z03092) was added and was then dialyzed against 2 L of NiA buffer overnight at 4 °C to reduce imidazole concentration. After dialysis, the eluate was then passed over fresh Ni-NTA agarose resin to orthogonally purify Pex15 with 6xHis tag removed by collecting the flowthrough.

### Thermal melt denaturation

Thermal stabilities of His-MBP-PSP-Pex1 D2 WT and G700D domains were monitored *via* increasing fluorescence of SYPRO Orange Protein Gel Stain (Sigma-Aldrich #200-664-3). SYPRO Orange Protein Gel Stain is naturally quenched in solution and fluoresces upon interaction with the hydrophobic core of a protein. The reaction mixture contained 1 mg/ml of purified MBP-fusion protein, 5× SYPRO Orange Protein Gel Stain, variable concentrations of ATP, and NiA. 0.5 M ATP was diluted in water to generate ATP solutions with the desired final concentration (0.5 mM, 1.5 mM, 2 mM, 2.5 mM, 3 mM, 5 mM, 7 mM, 8 mM, or 10 mM) in the protein reaction mixture. SYPRO Orange fluorescence of samples prepared in a 96-well plate were read over a temperature range of 10 °C to 95 °C, with 0.5 °C increments, in a melt curve protocol of a CFX96 Touch RT-PCR instrument (Bio-Rad). The negative derivative of relative fluorescence units (-d(RFU)/dt) was generated in GraphPad Prism and used to create the melt curves shown. The melting temperature (Tm) was determined using the “area under the curve” analysis in GraphPad Prism to calculate the minima of each melt curve. The minimum value of the concave up parabola corresponding to an unfolding event was determined as the melting temperature of a domain. Referenced temperature of a given domain is an average of the determined melting temperatures (N = 3) with the standard deviation shown for each. To determine the K_d_ of *Sc*PEX1^WT^ D2 domain binding to ATP, a one-site total nonlinear regression was calculated in GraphPad Prism and the value shown with 95% confidence interval.

### ATPase activity assay

The ATPase activity of WT and G700D *Sc*Pex1/*Sc*Pex6 complexes were monitored using an ATP/NADH-coupled enzymatic assay. In this assay, the regeneration of hydrolyzed ATP is coupled to the oxidation of NADH, which is measured at 340 nm (67). The reaction mixture contains 3 U/ml pyruvate kinase, 3U/ml lactate dehydrogenase, 1 mM NADH, and 7.5 mM phosphoenol pyruvate. The assays were performed in a 96-well plate and read on a SpectraMax M5 UV-visible plate reader spectrometer over 900 s with 10 s time points. For saturating conditions, we used 5 mM ATP. In each assay, the concentration of purified *Sc*Pex1/*Sc*Pex6 complexes used was to stay in the linear range throughout the duration of the experiment. For *Sc*Pex1^G700D^/*Sc*Pex6 complexes, this required higher concentrations ranging from 10 to 20 nM to achieve the same linearity. To determine the effect of *Sc*Pex15 inhibition, we preincubated *Sc*Pex1/*Sc*Pex6 complexes with *Sc*Pex15 (1–309) before addition to the ATPase assay reaction mixture.

### Fluorescein-5-maleimide unfolding assay

Substrate *Sc*Pex15 (1–309) protein was used at a final concentration of 2.5 μM, and each reaction included ATP regeneration mixture (ATP, creatine kinase, and creatine phosphate). *Sc*Pex15 (1-309) was diluted using buffer 1 (50 mM Hepes pH 7.5, 50 mM NaCl, 50 mM KCl, and 10 mM MgCl_2_) before the addition of Pex1/Pex6 complexes at a final concentration of 0.1 to 0.2 μM. The reaction mixture was incubated for 60 s to allow motor unfolding, then for an additional 30 s with 0.5 mM fluorescein-5-maleimide F5M (Thermo Fisher Scientific, #50-850-980) before quenching with equal volume of quench buffer (2% SDS and 10% β-mercaptoethanol). “Urea” samples were diluted in buffer 2 (50 mM Hepes pH 7.5, 50 mM NaCl, 50 mM KCl, and 10 mM MgCl_2_, and 8M urea) to a 6 M urea final concentration and incubated with F5M for 10 min before quenching. Excess F5M was run off SDS-PAGE gels, and the gels were imaged in the fluorescein channel (Bio-Rad ChemiDoc) prior to Coomassie-blue staining.

### Colorimetric peroxisome import assay

Import assays were performed as described previously (24). In brief, a Δ*pex1* deletion strain with the violacein pathway (VioA, VioB, and VioE-SKL) genomically incorporated at the *LEU2* locus was complemented with *CEN ARS* plasmids expressing *PEX1* variants. Three colonies from each transformation were grown overnight, diluted and grown to log-phase the next day, and spotted with 5 μl at *A*_600_ = 0.3 to evaluate the formation of green pigment. To quantify the fluorescence of prodeoxyviolacein metabolites, saturated cultures of yeast were lysed in acetic acid, filtered, and the fluorescence of the lysate was quantified at excitation 535 nm and emission 585 nm.

### Cell lines and culture

Primary and immortalized patient fibroblasts (a gift from the laboratory of Nancy Braverman, McGill University), HCT116, HEK293T, and HCT116 dCas9-KRAB (a gift from the laboratory of J. Corn, ETH Zürich) cells were cultured in Dulbecco’s modified Eagle’s media GlutaMax (Gibco, #10569010) supplemented with 10% fetal bovine serum (R&D Systems, #FS11150H) and 1% penicillin/streptomycin (Gibco, #15140122) and kept at 37 °C and 5% CO_2_ (patient fibroblasts were cultured at 7% CO_2_) in a humidified incubator. For routine passaging, adherent cells were grown to ∼70 to 80% confluency, washed with Dulbecco's phosphate buffered saline (DPBS) (Gibco, #14190-144), and subsequently treated with 0.25% trypsin-EDTA (Gibco, #25-200-072) for 3 to 5 min in a 37 °C incubator. Lifted cells were then quenched with DMEM and passaged. Supernatants from cell cultures and culture reagents in the lab are tested at random using the Invivogen MycoStrip *mycoplasma* detection kit (InvivoGen, #rep-mys), and *mycoplasma* contaminants were not identified.

HCT116 cells are human adult male colorectal carcinoma cells obtained from American Type Culture Collection. HCT116 dCas9-KRAB cells were selected for uptake of single guide RNA (sgRNA) plasmid with 1.5 μg/ml Puromycin (Gibco # A1113803) for 1 week and were continuously cultured in 1.0 μg/ml puromycin to maintain selection after this time.

For drug treatment conditions, cells were treated with 100 μg/ml CHX (Sigma-Aldrich, #C4859), 50 nM bafilomycin (Sigma-Aldrich, #B1793), 10 μM carfilzomib (Selleck, #PR-171) for between 1 and 24 h. Doxycycline treatments were either 1 μg/ml or 4 μg/ml doxycycline for tetON-PEX1^WT^ and tetON-PEX1^G843D^, respectively (Sigma-Aldrich, #D9891-5G) for 96 h.

### Genome editing with CRISPR-Cas9

HCT116 cells harboring epitope FLAG-tags were generated using CRISPR-Cas9 to introduce either N-terminal or C-terminal FLAG-tags, presence of the tag was verified in bulk culture, and then cells were dilution cloned and expanded. The presence and zygosity of FLAG insertion was confirmed with junction PCR, T7E1 digest, and Western blot. To introduce the G843D mutation, the FLAG-tagged HCT116 cells were edited at the PEX1 locus with CRISPR-Cas9, cells dilution cloned and expanded, and presence and zygosity of editing was confirmed with ClaI digests of PCR amplicons of the *PEX1* gene from exon 14 through exon 15 from genomic DNA. For CRISPR-Cas9 editing, cells were nucleofected by combining ribonucleoprotein buffer (100 mM Hepes, 750 mM KCl, 25 mM MgCl_2_, 25% glycerol, and 5 mM TCEP), 2.5 μM sgRNA, 2 μM Cas9 (purchased from Berkeley MacroLab), and 5 μM dsDNA donor, and then adding this mixture to 200k cells washed with DPBS (Gibco #14190-144) and resuspended in nucleofection buffer SE (Lonza #V4XC-1024). Reaction mixtures were then electroporated in 4D Nucleocuvettes (Lonza) and subsequently recovered with prewarmed media in culture dishes. The sequences of sgRNAs used in this work are as follows: PEX1 N terminus for FLAG epitope tag introduction: 5′-GCGACGCTCCGGGACGATGT-3′; PEX1 C terminus for FLAG epitope tag introduction: 5′-AGTGGAACAATGTTTCGACC-3′; PEX1 exon 15 for G843D introduction: 5′-TGGGTTGGGACAAGATTGGT-3′; nontargeting control for CRISPRi: 5′-GCTCCCGCGATCGGGGACA-3′; PEX1 for knockdown with CRISPRi: 5′-AGGCCGAGGACGTCGGAGC-3′; PEX6 for knockdown with CRISPRi: 5′-GACAGGACACCAACGAGGA-3′; UBR5 for knockdown with CRISPRi: 5′-CTGCGAGACGGAGAAACTG-3′; UBE2O for knockdown with CRISPRi: 5′-TTGGGCTCTGCGCGAGTCTCGGGGTTTAAGAGC-3′.

### RNA extraction, cDNA synthesis, and quantitative RT-PCR

Normalized cell counts were lysed with RLT buffer supplemented with 1% 2-mercaptoethanol and RNA was extracted using the Qiagen RNEasy kit (Qiagen #74106), according to the manufacturer's instructions. Genomic DNA in the cell lysate was presheared with Qiagen QIAshredder (Qiagen #79656) columns before RNA extraction. Subsequently, 1 μg of RNA was reverse transcribed to generate complementary DNA (cDNA) with the iScript Reverse Transcription Supermix (Bio-Rad, #1725035) according to the manufacturer’s protocol. RNA was stored at −80 °C, and cDNA was stored at −20 °C. The cDNA was diluted 10-fold in nuclease-free water and 2% was used as input for quantitative reverse transcription PCR using SYBR Green (Bio-Rad, #1725271) based gene-specific quantitative PCR (qPCR) in a CFX96 Touch qPCR instrument (Bio-Rad). All qPCR experiments were run with technical triplicates for each biological replicate and changes in gene expression were determined using the ΔΔCq method.

### Immunofluorescence staining

Cells were plated on glass bottom 96-well plates coated with either 50 μg/ml poly-D-lysine (Gibco, #A3890401) or Collagen (Corning, #354231) and fixed using 4% paraformaldehyde (Electron Microscopy Sciences, #15710) in DPBS (Gibco, #14190–144) for 10 min and washed with DPBS. Cells were then permeabilized using 0.1% Triton X-100 (Thermo Fisher Scientific, #A16046-AP) in DPBS for 10 min, blocked with buffer FZ (50 mM NH4Cl, 0.5% bovine serum albumin (BSA), 0.05% saponin, 0.02% NaN3 in PBS) for 20 min, and then probed with desired antibody diluted 1:1000 in buffer FZ overnight at 4 °C with gentle agitation. The following day, cells were washed 3× with DPBS-T and incubated with the appropriate fluorescently conjugated secondary antibody diluted 1:25,000 in buffer FZ. After secondary staining, 4′,6-diamidino-2-phenylindole staining (Invitrogen, #D1306) was carried out for 15 min in DPBS. Cells were then washed 3× in DPBS and stored in DPBS prior to image acquisition. All antibodies used for immunofluorescence staining in this study are as follows: Catalase (Proteintech, #66765-1-Ig), PMP70 (Invitrogen PA1-650), AlexaFluor 488 goat anti-rabbit IgG [H+L] (Invitrogen, #A11034), Goat anti-mouse IgG [H+L] Alexa Fluor 594 (Invitrogen #A32742). Antibodies for PMP70 and catalase were tested for peroxisomal localization specificity by immunostaining WT HCT116 cells expressing mNeonGreen-ZeoR-SKL (49). The PMP70 antibody was further validated by observing greatly reduced or no staining in PEX3 or PEX19 sgRNA expressing cells in which peroxisome membrane structures are lost.

### Confocal microscopy and analysis

Confocal microscopy images were acquired using an inverted spinning disc confocal microscope (Nikon Ti Eclipse) equipped with an electron multiplying charge-coupled device camera (Fusion SN:500241) and environmental control (Okolab stage top incubator). Image acquisition was performed with a 100× NA 1.64 oil-immersion objective using the NIS-Elements software. Exposure times and laser powers were standardized between replicates of an experiment. Each experiment was analyzed in technical triplicate, as well as biological triplicate. Sixteen images were collected for each technical replicate (*i.e.*, per well of a 96-well plate) in a fixed pattern. Raw micrographs were analyzed using the open-source image analysis software, CellProfiler. In brief, nuclei objects were identified and used to find cell borders by intensity-based edge propagation. PMP70 and catalase foci were identified, and their features quantified on a per cell basis. Pearson’s colocalization coefficient was calculated by the overlap of catalase positive pixels with PMP70 pixels and quantified on a per cell basis. All data presented contain colocalization coefficients calculated from N > 200 cells. Resulting analyses were plotted using Python 3.0.

### CHX chase assay

For all CHX chase experiments, 800k–1 million cells were seeded in 6 cm plates the day before treatment. For the autophagy inhibition experiments, cells were treated with either dimethyl sulfoxide, 50 nM bafilomycin-A1 (Cell Signaling Technology, #54645), or 50 nM bafilomycin-A1 and 100 μg/ml cycloheximide (Sigma-Aldrich, #C4859) at T = 0, and lysates were collected at indicated time points. For the proteasome inhibition experiments, cells were pretreated in 10 μM carfilzomib (Selleck, #PR-171) for 1 h before being released into media containing 100 μg/ml CHX and 10 μM carfilzomib at T = 0, and lysates were taken at each indicated time point. The half-life for CHX chase experiments were calculated with GraphPad Prism using one-phase decay nonlinear fit with 95% confidence interval shown.

### Lentiviral production and transduction and gene knockdown by CRISPRi

VSV-G pseudotyped lentiviral particles were generated by transfection of HEK293T cells with second generation lentiviral packaging vectors using TransIT-LT1 Transfection Reagent (Mirus #MIR2305) according to the manufacturer’s protocol. Lentivirus was harvested 72 h after transfection and stored at −80 °C. To perform transductions with the TetOn constructs, lentiviral supernatant was passively applied to cells overnight, followed by expansion and selection of clonal populations by fluorescence-activated cell sorting (Sony SH800S) for constitutive blue fluorescent protein expression. Clonal populations were then analyzed by flow cytometry (Attune NxT Flow Cytometer, Invitrogen) and populations expressing varying levels of blue fluorescent protein signal were expanded. Cells with high expression of TetOn-PEX1 were analyzed by treating cells with 2.0 μg/ml doxycycline for 24 h before analyzing expression *via* dot immunoblotting with the PEX1 antibody (BD Biosciences #611719).

Knockdown of candidate genes, PEX1, and PEX6 were performed using CRISPRi in the HCT116 dCas9-KRAB cell line. Constructs were made by cloning sgRNA sequences into the backbone vector pCRISPRia-v2, a gift from Jonathan Weissman (Addgene #84832). Lentivirus containing the candidate sgRNA were generated as described above and used to transduce the HCT116 dCas9-KRAB parental or PEX1^G843D^ cell line and selected for by 1.5 μg/ml Puromycin (Gibco, #A1113803) for at least 1 week. Gene knockdown was verified by reverse transcription quantitative polymerase chain reaction and/or immunoblotting.

### Immunoprecipitation

Cell lines were harvested at 15 to 20 million cells (normalized among conditions and replicates), spun 500*g* 5 min, washed in DPBS, and resuspended in LB1 (60 mM Hepes pH 7.6, 150 mM NaCl, 150 mM KCl, 10 mM MgCl_2_, 5% glycerol, 0.25 mM ATPγS (Biorbyt, #orb64057), 1.0% Triton X-100, and 0.5 mM EDTA) with added benzonase and 1× Protease Inhibitor (Halt, #PI78429). Cells were incubated on ice for 15 min, passed 5x through a 25G hypodermic needle to shear cells and genomic DNA, then incubated another 15 min on ice with mixing by inversion. The resulting lysate was then spun 15,000*g* 0 min at 4 °C and the supernatant was collected. Lysate was then precleared with Protein G agarose beads (EMD Millipore, #16-266), that were prewashed three times in LB1, for 30 min at 4 °C to reduce nonspecific binding and the beads were spun down 5000*g* 1 min and supernatant collected. Anti-FLAG M2 affinity agarose (Sigma-Aldrich #A2220) was washed three times in LB1, and then added to the precleared lysate and incubated for 2 to 3 h at 4 °C. The FLAG-agarose resin was washed three times in LB1, and twice with LB1 containing no detergent. Proteins were eluted with 300 μg/ml FLAG-peptide (Sigma-Aldrich #F3290) dissolved in LB1 with no detergent by incubating for 30 min shaking at 1100 rpm at 4 °C. Beads were spun 5000g × 1 min and eluates were collected. Eluates were snap-frozen in liquid nitrogen and stored at −80 °C.

### MS sample preparation

FLAG coimmunoprecipitation eluates were prepared for MS by first precipitating proteins with 20% TCA at −80 °C overnight. The protein pellets were washed three times in ice-cold solution of 0.01 N HCl in 90% acetone and air-dried. Pellets were then resuspended in 100 mM Tris pH 8.5, 8 M urea, and 3 mM TCEP (GoldBio #51805-45-9) was added and incubated for 20 min at room temperature. Then, 10 mM iodoacetamide (Acros Organics #144-48-9) was added and incubated for 15 min at room temperature in the dark. Samples were then diluted 4-fold with 100 mM Tris pH 8.5, and 1 mM CaCl_2_ was added along with 1 μg of Trypsin (Promega #V5280) and incubated overnight at 37 °C. Digested protein samples were then snap-frozen in liquid nitrogen and sent to UC Davis Proteomics Core Facility for analysis.

### MS (LC-MS/MS)

Liquid chromatography (LC) was performed on an ultra-high pressure LC system, nanoElute (Bruker Daltonics), at 40 °C and with a constant flow of 400 nl/min on a PepSep 150 μm × 25 cm C18 column (PepSep) with 1.5 μm particle size (100 Å pores) (Bruker Daltonics), and a ZDV spray emitter (Bruker Daltonics). Mobile phases A and B were water with 0.1% formic acid (v/v) and 80/20/0.1% acetonitrile/water/formic acid (v/v/v), respectively. Peptides were separated using a 60 min gradient.

MS was performed on a hybrid trapped ion mobility spectrometry-quadrupole time-of-flight mass spectrometer (timsTOF pro, Bruker Daltonics) with a modified nano-electrospray ion source (CaptiveSpray, Bruker Daltonics). The mass spectrometer was operated in parallel accumulation–serial fragmentation (PASEF) mode. Desolvated ions entered the vacuum region through the glass capillary and deflected into the trapped ion mobility spectrometry (TIMS) tunnel which is electrically separated into two parts (dual TIMS). The first region is operated as an ion accumulation trap that primarily stores all ions entering the mass spectrometer, while the second part performs trapped ion mobility analysis.

A single TIMS-MS scan is composed of many individual TOF scans of about 110 ms each. All experiments were acquired with a 100 ms ramp and 10 PASEF tandem mass spectrometry (MS/MS) scans per topN acquisition cycle. In TOF MS, signal-to-noise ratios can conveniently be increased by summation of individual TOF scans. Here, low-abundance precursors with an intensity below a “target value” were repeatedly scheduled for PASEF-MS/MS scans until the summed ion count reached the target value (*e.g.* four times for a precursor with the intensity 5000 arbitrary units (a.u.) and a target value of 20,000 a.u. We set the target value to 20,000 a.u. for all methods. MS and MS/MS spectra were recorded from m/z 100 to 1700. A polygon filter was applied to the m/z and ion mobility plane to select features most likely representing peptide precursors rather than singly charged background ions. The quadrupole isolation width was set to 2 Th for m/z under 700 and 3 Th for m/z larger than 700, and the collision energy was ramped stepwise as a function of increasing ion mobility: 52 eV for 0 to 19% of the ramp time; 47 eV from 19 to 38%; 42 eV from 38 to 57%; 37 eV from 57 to 76%; and 32 eV for the remainder.

### MS data analysis

All MS/MS samples were analyzed using MSFragger (68). MSFragger was set up to search the 2021-11-16-decoys-contam-UP000005640 database assuming the digestion enzyme strict trypsin. MSFragger was searched with a fragment ion mass tolerance of 20 PPM and a parent ion tolerance of 20 PPM. Carbamidomethyl of cysteine was specified in MSFragger as a fixed modification. Oxidation of methionine and acetyl of the N terminus were specified in MSFragger as variable modifications.

Criteria for protein identification: Scaffold (version Scaffold_5.0.1, Proteome Software Inc., Portland, OR) was used to validate MS/MS based peptide and protein identifications. Peptide identifications were accepted if they could be established at greater than 0.0% probability by the PeptideProphet algorithm (69). Protein identifications were accepted if they could be established at greater than 20.0% probability and contained at least two identified peptides. Protein probabilities were assigned by the ProteinProphet algorithm (70). Proteins that contained similar peptides and could not be differentiated based on MS/MS analysis alone were grouped to satisfy the principles of parsimony. Proteins sharing significant peptide evidence were grouped into clusters.

### Transient transfection

Transient transfection plasmids containing the PEX1^G843D^-OTUB1 fusions were cloned by Gibson assembly to insert PEX1^G843D^ into the transient transfection OTUB1 or OTUB1^C91S^ pcDNA3.1 plasmids (Addgene #118209 and Addgene #118210, respectively). FLAG-PEX1^G843D/G843D^ cells were transfected with the PEX1^G843D^-OTUB1 fusion plasmids using Mirus TransIT-LT1 (Mirus #MIR2305) in 10-cm plates 48 h prior to treatment with CHX. Briefly, 24 h prior to CHX treatment, transfected cells were lifted and seeded into 6-cm plates for each time point. Cells were then treated with 100 μg/ml CHX at T0 and harvested at the indicated time points.

### Immunoblotting

Normalized cell counts were lysed in either (1) radioimmunoprecipitation assay buffer containing benzonase (Millipore Sigma, #70664) and protease inhibitor cocktail (Halt, #PI78429) for 30 min on ice before combining with 2× Laemmli sample buffer containing 2-mercaptoethanol or (2) directly in 2× Laemmli sample buffer containing 2-mercaptoethanol before sonicating for 30 s at 50% amplitude (Branson) and boiling for 5 min at 95 °C. For the CHX chase experiments, cells were lysed with 2× Laemmli sample buffer with 2-mercaptoethanol directly in culture plates and scraped to collect cells. Samples were resolved with either 4 to 20% SDS-PAGE gels (Bio-Rad, #4561095) or homemade bis-Tris Gels. Proteins were transferred to 0.45 μm LF polyvinylidene fluoride membranes using semidry (Bio-Rad) or wet-transfer methods with Towbin transfer buffer (192 mM Glycine, 25 mM Tris-base, 20% methanol). Membranes were blocked in 3% BSA (Thermo Fisher Scientific, #BP1605–100) in TBST (Tris-buffered saline with 0.1% Tween-20) for 1 h, and then probed with desired primary antibody in 3% BSA in TBST overnight at 4 °C with gentle agitation. The next day membranes were washed 3 x 5 min each with TBST before probing with species-specific horseradish peroxidase-conjugated secondary antibodies dissolved in 5% milk or 3% BSA in TBST for 1 h. Membranes were washed 3 x 5 min each with TBST before visualization of chemiluminescence using either Pierce ECL2 Western Blotting Substrate (Thermo Fisher Scientific, #PI80196) or Pierce SuperSignal West Femto Substrate (Thermo Fisher Scientific, #34095) on a ChemiDoc MP imaging system (Bio-Rad). The antibodies used for immunoblotting in this study are as follows: PEX1 (BD Biosciences, #61171), FLAG M2 (Sigma-Aldrich, #F1804), LC3I-II (Abcam, #ab192890), HIF1α (BD Biosciences, #610958), SCP2 (Sigma-Aldrich, #HPA027317), PEX6 (Santa Cruz Biotechnology, #sc-271813). The PEX1, PEX6, and HIF1⍺ antibodies were validated by immunoblotting cell lysates from cultures expressing either nontargeting control sgRNAs, or sgRNAs specific to PEX1, PEX6, or HIF1⍺. The FLAG antibody was validated using FLAG peptide as a positive control. The antibody for LC3I-II was validated by the observation of LC3II accumulation upon bafilomycin A1 treatment, characteristic of failed autophagosome maturation. The antibody for SCP2 was validated by the disappearance of the mature SCP2 protein (46 kDa) upon disruption of peroxisome import by PEX1 or PEX6 sgRNA expression.

### Densitometry

Western Blot images were quantified by pixel densitometry using FiJi. All analyses were performed on raw unadjusted images. All analyses presented are normalized to the total pixel density in the lane observed in the Stain-Free blot image, representing total protein load in each lane. Stain-Free gel technology provides a greater linearity and wider dynamic range for total protein quantifications as a loading control compared to housekeeping gene controls (71).

### Statistical analyses

All statistical analyses were performed using GraphPad Prism and descriptive statistics are described in figure legends where relevant.

## Data availability

The MS proteomics data have been deposited to the ProteomeXchange Consortium (72) *via* the PRIDE (73) partner repository with the dataset identifier PXD061295 and 10.6019/PXD061295.

## Supporting information

This article contains supporting information.

## Conflict of interest

The authors declare that they have no conflicts of interest with the contents of this article.
